# Lysosomal Acid Lipase Deficiency: A Report of Two Cases and a Review of the Literature

**DOI:** 10.7759/cureus.73299

**Published:** 2024-11-08

**Authors:** Calin Nedelcu, Irina Dijmarescu, Marina Patrascoiu, Ioana Oprescu, Daniela Pacurar

**Affiliations:** 1 Pediatrics, “Grigore Alexandrescu” Emergency Children’s Hospital, Bucharest, ROU; 2 Pediatrics, “Carol Davila“ University of Medicine and Pharmacy, Bucharest, ROU

**Keywords:** cholesteryl ester storage disease, lal-d, lysosomal acid lipase deficiency, pediatric patients, wolman disease

## Abstract

Lysosomal acid lipase deficiency (LAL-D) is an autosomal recessive genetic disease arising from mutations in the lipase A, lysosomal acid type (LIPA) gene, characterised by the formation of cholesterol esters and triglyceride storages, primarily in the liver and spleen. By analysing the level of lysosomal acid lipase (LAL), two forms were described in the literature: Wolman disease and cholesteryl-ester storage disease (CESD).

Wolman disease usually manifests with rapidly progressive symptoms within the first year of life, while CESD is a latent condition, with significant features appearing later in life. LAL-D usually presents with a non-specific tableau, thus having the possibility of being incorrectly labelled. LAL activity can be demonstrated by LAL dried blood spot (DBS) testing. Since 2015, sebelipase alfa has been authorised for the treatment of LAL-D in the European Union, changing the course of this disease. This article aims to present two clinical cases of CESD and review the current literature on LAL-D.

We presented two cases of CESD who were evaluated in our department for chronically elevated transaminases, one with and one without dyslipidaemia. For both patients, other causes of chronic hepatitis were excluded (viral infections, autoimmune diseases, and metabolic diseases), and LAL DBS testing was positive, establishing the diagnosis of CESD. One of them also underwent genetic testing, and a homozygous mutation of the LIPA gene was identified.

LAL-D is a difficult diagnosis to establish, considering the scarcity of knowledge. In addition, neither the LAL DBS test nor LIPA gene sequencing investigations are largely available. Thus, most asymptomatic or minimally symptomatic patients might remain undiagnosed, and multiple complications without clear aetiology might arise, making this disease even harder to diagnose and manage. Nevertheless, even though the introduction of enzyme replacement therapy has improved outcomes for many patients, there still remains a need for increased awareness and understanding of this rare condition among health professionals.

## Introduction

Lysosomal acid lipase deficiency (LAL-D) is an autosomal recessive genetic disease, arising from mutations in the lipase A, lysosomal acid type (LIPA) gene, characterised by the formation of cholesterol esters (CEs) and triglyceride (TG) storages, primarily in the liver and spleen, but also in the adrenal glands, lymph nodes, gastrointestinal tract, blood vessels, and skeletal muscle tissue. By analysing the level of lysosomal acid lipase (LAL), two forms were described in the literature: Wolman disease (complete absence of LAL) and cholesteryl-ester storage disease (CESD) (partial deficiency of LAL) [1,2]. This article aims to present two clinical cases of CESD, a rare, underdiagnosed disease, and review the current literature on LAL-D.

LAL-D presents with a wide spectrum of symptoms on a multisystemic level. Wolman disease usually manifests with rapidly progressive symptoms within the first 12 months of life, while CESD is a latent condition, with more variable and milder symptoms appearing later in life [1]. Typically, infants present with severe vomiting in the first month of life, watery stools (with or without steatorrhea), mild fever, and severe malnutrition. The particular aspect found on imaging is enlargement of the adrenal glands, often with punctate calcifications [1,2]. Signs or symptoms of adrenal insufficiency are often associated, together with abdominal distension secondary to hepato-splenomegaly. As the disease progresses, other elements may appear in relation to the chronic evolution, such as anaemia, jaundice, and cachexia [2].

## Case presentation

The first patient, an eight-year-old female patient, presented to our department for evaluation with chronic elevated transaminases and dyslipidaemia. She was first identified approximately five years prior, during a hospital admission, when she was diagnosed with Epstein-Barr virus infection. Two years later, routine laboratory tests showed persistently high liver enzymes. The personal history of the patient was unremarkable, but family history revealed that her father had similar laboratory findings. Ultrasound, FibroScan, and FibroTest were normal. Autoimmune hepatitis, Wilson disease, and coeliac disease were excluded.

After another two and a half years, she presented for reassessment. Clinical evaluation showed mild malnutrition (24 kg, 135 cm, with a body mass index of 13.2 kg/m2 and a corresponding Z-score of -1.72 standard deviations according to the World Health Organization) with no clinical signs of dyslipidaemia or hepatic illness.

Laboratory tests showed elevated liver enzymes, no cholestasis (normal bilirubinaemia, alkaline phosphatase, and gamma-glutamyl transferase), and no coagulopathy. The lipid metabolism evaluation described low high-density lipoprotein-cholesterol (HDL-C) and high values for low-density lipoprotein-cholesterol (LDL-C), very-low-density lipoprotein-cholesterol (VLDL-C), total lipids, and total TGs (Table 1).

**Table 1:** Initial hepato-billiary assessment – laboratory tests (patient 1) ALT: alanine transferase, AST: aspartate transferase, GGT: gamma-glutamyl transferase, AF: alkaline phosphatase, HDL-C: high-density lipoprotein-cholesterol, LDL-C: low-density lipoprotein-cholesterol, VLDL-C: very-low-density lipoprotein-cholesterol, TGs: triglycerides, aPTT: activated partial thromboplastin time, PT: prothrombin time, INR: international normalized ratio

Test name	Result	Normal range
ALT	87 U/L	0-35 U/L
AST	65 U/L	0-35 U/L
GGT	12 U/L	4-20 U/L
AF	289 U/L	86-315 U/L
Conjugated bilirubin	0.09 mg/dL	0.0-0.2 mg/dL
Unconjugated bilirubin	0.38 mg/dL	0-1 mg/dL
Total bilirubin	0.47 mg/dL	0.3-1.2 mg/dL
HDL-C	37 mg/dL	>40 mg/dL
LDL-C	290 mg/dL	0-100 mg/dL
VLDL-C	54 mg/dL	2-30 mg/dL
Total lipids	1248 mg/dL	400-800 mg/dL
Total TGs	271 mg/dL	0-150 mg/dL
aPTT	32.6 sec	24.4-36.4 sec
PT	13.4 sec	11.0-14.0 sec
INR	1.21	0.80-1.3

A broader differential diagnosis for chronic cytolysis was pursued, including infections (hepatitis B virus and hepatitis C virus), autoimmune liver disease (normal values of gamma-globulins and immunoglobin G, negative specific autoantibodies titers), Wilson disease (normal ceruloplasmin level), coeliac disease (negative titers of anti-tissue transglutaminase antibodies), and metabolic diseases (Niemann-Pick, Gaucher, Wolman, glicogenoses, and congenital fructose intolerance) (normal liver and spleen sizes, lack of symptoms for age, normal blood glucose, and no reaction to fructose intake). Low activity on the LAL dried blood spot (DBS) test was identified (<0.02 nmol/punch/h, with a normal range between 0.37 and 2.3), and the patient was diagnosed with CESD (Table 2). The patient’s parents did not approve the initiation of the enzyme-replacement therapy, and the patient was lost to follow-up.

**Table 2:** Differential diagnosis for chronic liver cytolysis – laboratory tests (patient 1) HBs Ag: hepatitis B surface antigen, anti-HCV Ab: anti-hepatitis C virus antibodies, IgG: immunoglobulins G, anti-LKM-1 Ab: anti-liver kidney microsomal type 1 antibodies, ASMA: anti-smooth muscle antibodies, IgA: immunoglobulins A, anti-TTG IgA Ab: anti-tisular transglutaminase IgA antibodies, LAL DBS: lysosomal acid lipase dried blood spot

Test name	Result	Normal range
HBs Ag	Negative	Negative
Anti-HCV Ab	Negative	Negative
Gamma-globulins	14.1%	11-22%
IgG	12.83	5-13 g/L
Anti-LKM-1 Ab	3.03 U/mL	<4 U/mL
ASMA	10.03 U/mL	0-20 U/mL
Ceruloplasmin	23 mg/dL	16-45 mg/dL
IgA	1.45 g/L	0.48-3.45 g/L
Anti-TTG IgA Ab	4.2 U/mL	<10 U/mL
Blood glucose	71 mg/dL	60-100 mg/dL
LAL DBS test	<0.02 nnmol/punch/h	0.37-2.3 nmol/punch/h

The second patient, an eight-year-old boy, was referred to our hospital for enzyme replacement therapy. At age two, he was evaluated in another hospital for persistently elevated liver enzymes. The abdominal ultrasonography described hepatic steatosis, and the patient was further evaluated by FibroScan (F0 stage, Metavir). The patient was evaluated for viral infections (negative serologies), autoimmune liver disease (no hypergammaglobulinemia, normal IgG, negative autoantibodies), Wilson disease (normal ceruloplasmin), coeliac disease (negative anti-TTG Ab), and metabolic diseases. The LAL DBS was positive (<0.03 nmol/punch/h, with a normal range between 0.37 and 2.3), followed by a genetic dyslipidaemia panel that discovered a homozygous mutation of the LIPA gene (c.894G>A, silent) (Table 3). The diagnosis of CESD was confirmed.

**Table 3:** Hepato-biliary assessment and differential diagnosis for chronic cytolysis – laboratory tests (patient 2) ALT: alanine transferase, AST: aspartate transferase, HDL-C: high-density lipoprotein-cholesterol, LDL-C: low-density lipoprotein-cholesterol, TGs: triglycerides, HBs Ag: hepatitis B surface antigen, anti-HCV Ab: anti-hepatitis C virus antibodies, IgG: immunoglobulin G, anti-LKM-1 Ab: anti-liver kidney microsomal type 1 antibodies, ASMA: anti-smooth muscle antibodies, IgA: immunoglobulin A, anti-TTG IgA Ab: anti-tisular transglutaminase IgA antibodies, LAL DBS: lysosomal acid lipase dried blood spot

Test name	Result	Normal range
ALT	77 U/L	0-35 U/L
AST	73 U/L	0-35 U/L
Conjugated bilirubin	0.11 mg/dL	0.0-0.2 mg/dL
Unconjugated bilirubin	0.18 mg/dL	0-1 mg/dL
Total bilirubin	0.29 mg/dL	0.3-1.2 mg/dL
HDL-C	58 mg/dL	>40 mg/dL
LDL-C	92.8 mg/dL	0-100 mg/dL
Total TGs	77 mg/dL	0-150 mg/dL
HBs Ag	Negative	Negative
Anti-HCV Ab	Negative	Negative
Gamma-globulins	16.3%	11-22%
IgG	10.22	5-13 g/L
Anti-LKM-1 Ab	2.80 U/mL	<4 U/mL
ASMA	4.56 U/mL	0-20 U/mL
Ceruloplasmin	34 mg/dL	16-45 mg/dL
IgA	2 g/L	0.48-3.45 g/L
Anti-TTG IgA Ab	5.3 U/mL	<10 U/mL
Blood glucose	64 mg/dL	60-100 mg/dL
LAL DBS test	<0.03 nnmol/punch/h	0.37-2.3 nmol/punch/h

The patient was started on sebelipase alfa 1 mg/kg/dose. Due to the lack of response (persistently elevated liver enzymes), the dose was elevated to 2 mg/kg/dose. No adverse effects were reported. He is currently at the 15th dose of treatment, administered every other week. During follow-up, he was being monitored for abnormal values in complete blood count (CBC), aspartate transferase (AST), alanine transferase (ALT), gamma-glutamyl transferase (GGT), alkaline phosphatase, lipid profile (TGs, total cholesterol, total lipids), and kidney function tests (urea, creatinine), presenting normal values for AST and ALT after the increased dose of treatment.

## Discussion

**Figure 1:**
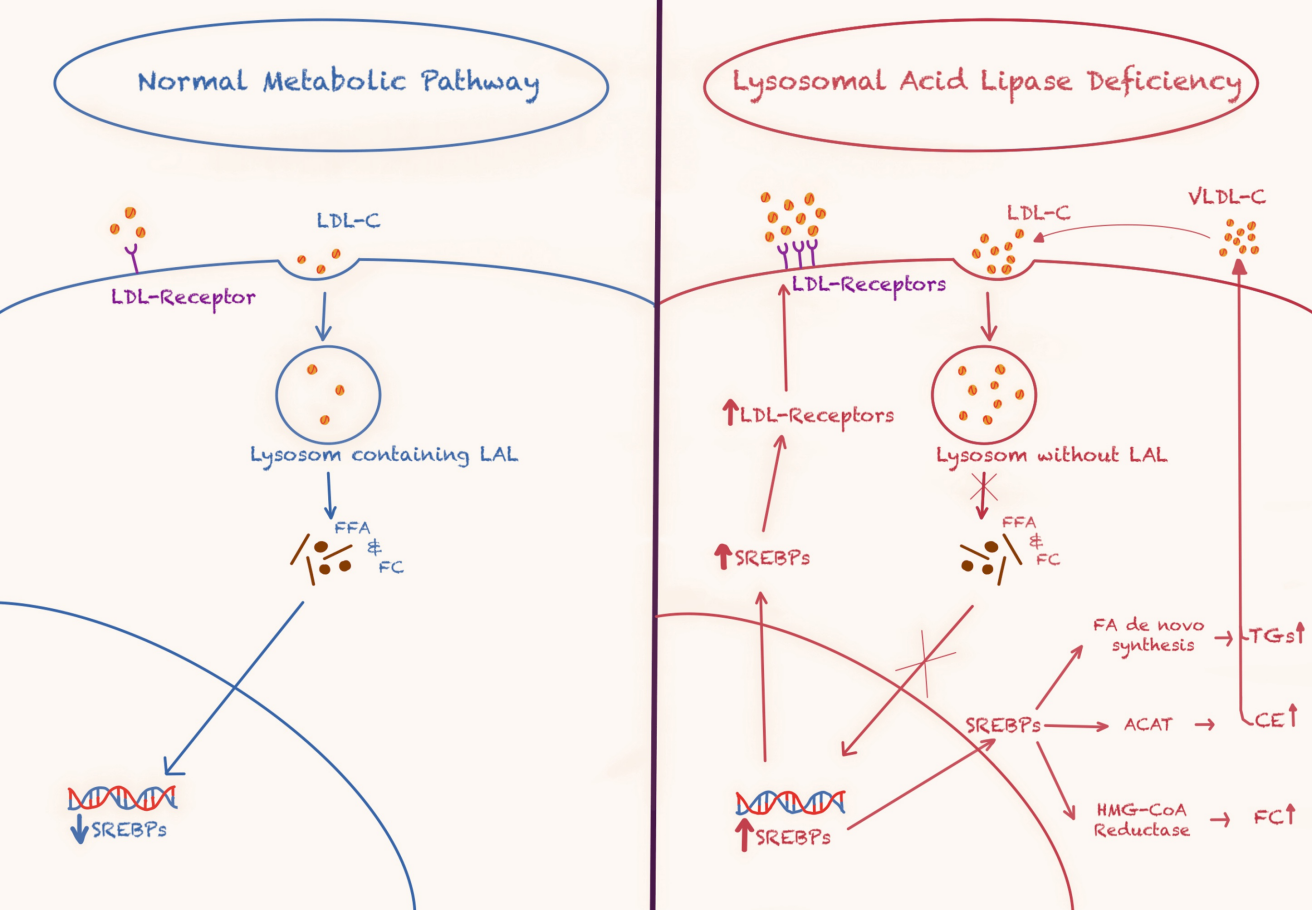
LDL-C metabolic pathway in healthy individuals and patients suffering from LAL-D ACAT: acyl-CoA cholesterol acyltransferase, LDL-C: low-density lipoprotein-cholesterol, LAL-D: lysosomal acid lipase deficiency, LDL: low-density lipoprotein, LAL: lysosomal acid lipase, FFA: free fatty acid, FC: free cholesterol, SREBPs: sterol regulatory-element binding proteins, VLDL-C: very-low-density lipoprotein-cholesterol, FA: fatty acid, CE: cholesterol ester, HMG-CoA: hydroxymethylglutaryl-coenzyme Image Credit: Created by the first author, Calin Nedelcu, based on Sheth et al. (2023) [4]

LAL is a hydrolase that degrades the CEs and TGs from the lipoproteins endocytosed in the lysosome, forming free cholesterol (FC) and free fatty acids (FFA). LAL is found in every type of cell except for erythrocytes and helps regulate intracellular lipid metabolism. The presence of cytosolic FC downregulates the activation of sterol regulatory element-binding proteins, which controls the production of new molecules of cholesterol through 3-hydroxy-3-methylglutaryl coenzyme A reductase, an enzyme responsible for the first step in the metabolic pathway that produces cholesterol, and the expression of low-density lipoprotein receptors on the surface, thus reducing the endocytosis of LDL. The absence of LAL makes the cell think that the body does not have enough serum cholesterol and activates the production of de novo FFA for reforming TGs and phospholipid molecules for new VLDL molecules [3,4]. These changes in the metabolic pathway are depicted in Figure 1.

Currently, the real prevalence of LAL-D is unknown, considering that it is a rare disease, often underdiagnosed or unreported. The actual estimate ranges between 1:40.000 and 1:300.000, depending on the ethnicity and geographical location. This prevalence rate was estimated only based on the E8SJM mutation of the LIPA gene, which is responsible only for 50-70% of the cases of LAL-D in diagnosed children and adults [2]. Some other studies report different prevalence rates in specific cohorts, such as Germans (1:50.000 for CESD and 1:350.000 for Wolman disease) or Iranian-Jewish individuals from the Los Angeles area (1:4.200) [5,6].

Symptoms of Wolman disease can be observed shortly after birth, while CESD most commonly becomes evident before the age of five, but there are cases reported being diagnosed as late as the seventh decade of life [7,8]. Nevertheless, even when CESD was diagnosed later in life, a thorough review of clinical records often revealed that signs and symptoms were present from early childhood [9].

A review that analysed 39 case reports including a total of 71 patients found the following features: hepatomegaly and splenomegaly (85%), gastrointestinal symptoms, such as vomiting, diarrhoea, failure to thrive, abdominal pain, and gastrointestinal bleeding (30%), anaemia (14%), gallbladder dysfunctions (4%), pulmonary symptoms (3%), and atherosclerosis (1,5%) [8]. Some observational and case report studies described cardiovascular events at a young age due to arterial plaque and atheroma, but these observations are not yet thoroughly examined and, in practice, are far less recognised as opposed to gastroenterological complications [1]. Moreover, seven patients aged between 27 and 58 years old, reported until 2000, presented with minimal to no symptoms, uncharacteristic presentations, or pathologies that diverged the diagnostic process. From these, we list minor dyspeptic symptoms, high cholesterol as the sole sign of illness, stroke, liver cancer, marked hepatic fibrosis, or decompensated cirrhosis [10-12].

Our patients presented with no symptoms and minimal to no signs of disease, needing thorough evaluations and multiple laboratory tests. A high level of suspicion was necessary, especially for the eight-year-old female patient. Both the patient and her father had a similar presentation, not corresponding with an autosomal recessive pattern. However, genetic testing confirmed the diagnosis.

Taking into consideration the scarce previous knowledge, it is safe to assume that several asymptomatic and minimally symptomatic patients might still be undiagnosed. In consequence, multiple complications without clear aetiology might have arisen, making this disease even harder to diagnose and treat. To a broad spectrum of medical specialists, such as paediatricians, gastroenterologists, endocrinologists, cardiologists, nephrologists, and general practitioners, it is of utmost importance to be aware of the multiple facets that this ailment can present.

Typically, laboratory tests show elevated total cholesterol, elevated LDL-C, low HDL-C, and elevated transaminases. Even though liver biopsy is not required for the diagnosis, particular histological aspects, such as foamy macrophages and microvesicular steatosis, have been described in the literature. Moreover, the presence of birefringent crystals of CEs, markedly enlarged Kupffer cells, and macrophages strongly receptive to periodic acid-Schiff staining or the detection of lysosomal-associated membrane protein 1, lysosomal-associated membrane protein 2, and cathepsin D on immunohistochemical samples should raise suspicion and point to a diagnosis of LAL-D [1,2].

Historically, the enzyme activity was measured in cultures of fibroblasts, leukocytes, or liver tissue. However, the substrates used were not specific enough, being prone to generating false-negative results. LAL activity can be demonstrated by LAL DBS testing using an inhibitor specific for LAL called Lalistat 2. The results are obtained by comparing the degradation of lipidic molecules before and after the inactivation of LAL, with a normal range between 0.37 and 2.3 nmol/punch/h. For borderline values, the test is considered inconclusive, thus requiring LIPA gene sequencing. Unfortunately, neither the LAL DBS test nor LIPA gene sequencing are largely available. Twelve laboratories in only eight countries (Argentina, Brazil, France, Germany, Italy, Japan, the United Kingdom, and the United States) can examine LAL DBS test samples [2].

LAL-D usually presents with a non-specific tableau, thus being incorrectly labelled as heterozygous familial hypercholesterolaemia, familial combined hyperlipidaemia, polygenic hypercholesterolaemia, non-alcoholic fatty liver disease, non-alcoholic steatohepatitis, or cryptogenic liver disease. Firstly, to ensure the best clinical practice, a thorough family history and screening for viral and autoimmune hepatitis, as well as for metabolic diseases, should be conducted. Nevertheless, any non-obese patient with abnormal laboratory values as listed above should be screened for LAL-D [2].

Until recently, treatment options for LAL-D only consisted of dietary changes and hypolipidemic medication. Several procedures were used to cope with the rapid evolution of this disease. Splenectomy, ligation of the esophageal varices, and liver or haematopoietic stem cell transplantation were all ideas that failed to deliver a satisfactory outcome, burdening the patients with numerous complications [13]. Since August 2015, sebelipase alfa has been authorised for the treatment of LAL-D in the European Union, changing the course of this disease. This recombinant human enzyme-replacement therapy for LAL-D, administered intravenously every other week, is now the only approved treatment [14].

The most common adverse effects encountered in infants were allergic reactions, palpebral oedema, tachycardia, dyspnoea, vomiting, diarrhoea, and fever. For older children, the following were classified as very common (allergic reactions, drowsiness, abdominal pain, diarrhoea, fatigue, and fever) and as common (tachycardia, hypotension, dyspnoea, and abdominal distension) [14]. In addition, there are case reports in the literature focusing on patients with a suboptimal response to enzyme-replacement therapy or patients who manifested allergic reactions and required desensitisation [15,16].

Regarding complications, the most commonly encountered are liver cirrhosis, hepatic carcinoma, and cardiovascular complications. Currently, new research suggests that patients suffering from LAL-D are also more prone to life-threatening conditions (such as hemophagocytic lymphohistiocytosis) or have a higher odds ratio of all-cause fractures [17,18].

Given that the enzyme deficiency has primary effects on the hepatic and cardiovascular systems, the following tests are recommended for follow-up: CBC, AST, ALT, GGT, alkaline phosphatase, and kidney function tests, consisting of urea, creatinine levels, and estimated glomerular filtration rate. The proposed course of action states that the lipidic profile should consist of total cholesterol, TGs, and HDL-C. Only when TG values are over 400 mg/dL (4.52 mmol/L), direct LDL-C should be requested. A non-invasive hepatic assessment should be performed annually. Furthermore, a cardiovascular assessment is recommended between one and two years for patients with atherosclerosis and between two and five years in the absence of symptoms [19].

This article summarises and highlights key aspects of this disease while presenting two almost asymptomatic patients who have been diagnosed with CESD. As for the limitations, none of the patients have been completely investigated in our department. One of them did not proceed with the proposed treatment plan, while the other was referred to us after being diagnosed. Also, for both patients, the disease is ongoing and it may evolve in time.

## Conclusions

LAL-D is a complex disorder that requires a high degree of clinical suspicion for diagnosis, mainly for non-obese patients with unexplained dyslipidaemia and liver enzyme abnormalities. While the introduction of enzyme-replacement therapy has improved outcomes for many individuals, there still remains a need for increased awareness and understanding of this rare condition among healthcare professionals.

Systematic reviews analysing presenting symptoms, complications, and clinical trials focused on dosage, frequency of administration, and potential adverse effects may be beneficial for improving patient care in LAL-D. As for improved clinical judgement, better and more nuanced diagnostic protocols need to be developed, considering the economic and psychological burden our patients face.
